# Global phylogenomic novelty of the Cas1 gene from hot spring microbial communities

**DOI:** 10.3389/fmicb.2022.1069452

**Published:** 2022-12-02

**Authors:** Oscar Salgado, Sergio Guajardo-Leiva, Ana Moya-Beltrán, Carla Barbosa, Christina Ridley, Javier Tamayo-Leiva, Raquel Quatrini, Francisco J. M. Mojica, Beatriz Díez

**Affiliations:** ^1^Department of Molecular Genetics and Microbiology, Biological Sciences Faculty, Pontifical Catholic University of Chile, Santiago, Chile; ^2^Núcleo de Ciencias Naturales y Exactas, Universidad Adventista de Chile, Chillán, Chile; ^3^Departamento de Microbiología, Universidad de Talca, Talca, Chile; ^4^Centro de Ecología Integrativa, Universidad de Talca, Talca, Chile; ^5^Centro Científico y Tecnológico de Excelencia Ciencia & Vida, Santiago, Chile; ^6^Departamento de Geología, Facultad de Ciencias Físicas y Matemáticas, Universidad de Chile, Santiago, Chile; ^7^Centro de Excelencia en Geotermia de Los Andes (CEGA-Fondap), Santiago, Chile; ^8^Facultad de Medicina y Ciencia, Universidad San Sebastián, Santiago, Chile; ^9^Departamento de Fisiología, Genética y Microbiología, Universidad de Alicante, Alicante, Spain; ^10^Center for Climate and Resilience Research (CR)^2^, Santiago, Chile; ^11^Millennium Institute Center for Genome Regulation (CGR), Santiago, Chile

**Keywords:** Cas1, hot spring, phylogenomic, CRISPR-Cas, casposase

## Abstract

The Cas1 protein is essential for the functioning of CRISPR-Cas adaptive systems. However, despite the high prevalence of CRISPR-Cas systems in thermophilic microorganisms, few studies have investigated the occurrence and diversity of Cas1 across hot spring microbial communities. Phylogenomic analysis of 2,150 Cas1 sequences recovered from 48 metagenomes representing hot springs (42–80°C, pH 6–9) from three continents, revealed similar ecological diversity of Cas1 and 16S rRNA associated with geographic location. Furthermore, phylogenetic analysis of the Cas1 sequences exposed a broad taxonomic distribution in thermophilic bacteria, with new clades of Cas1 homologs branching at the root of the tree or at the root of known clades harboring reference Cas1 types. Additionally, a new family of casposases was identified from hot springs, which further completes the evolutionary landscape of the Cas1 superfamily. This ecological study contributes new Cas1 sequences from known and novel locations worldwide, mainly focusing on under-sampled hot spring microbial mat taxa. Results herein show that circumneutral hot springs are environments harboring high diversity and novelty related to adaptive immunity systems.

## Introduction

Adaptive immunity in *Bacteria* and *Archaea* is achieved by CRISPR-Cas (clustered regularly interspaced short palindromic repeats and CRISPR-associated genes) systems (Mohanraju et al., 2016; Makarova et al., 2020a). When a foreign nucleic acid invades a prokaryotic cell, it can be recognized by adaptation Cas proteins generating small fragments (spacers) that are stored in a CRISPR array of the host genome separated by the repeats (McGinn and Marraffini, 2019). This information establishes an immunological memory in these cells because spacer-containing transcripts will guide effector Cas to cleave the invasive nucleic acid in future encounters. In most cases, the invading nucleic acid corresponds to viruses (Shmakov et al., 2017a). Four modules, composed of different Cas, have been defined in the functioning of CRISPR-Cas systems (Makarova et al., 2015). The adaptation module integrates the spacers into the host through a complex formed by Cas1 and Cas2, assisted by non-Cas and sometimes other Cas proteins (e.g., Cas4, Cas3, or Cas9) (Koonin and Krupovic, 2015; Amitai and Sorek, 2016; Jackson et al., 2017). Subsequently, the expression module processes multi-spacer transcripts from the CRISPR array (pre-crRNA) to deliver single spacer-containing RNA fragments (crRNAs) that guide Cas of the interference module to act against the invading nucleic acid recognized through complementary bases-pairing with the spacer sequences (Hille et al., 2018). Finally, several proteins or domains in the ancillary/helper module have been described as playing accessory roles (Makarova et al., 2020a). CRISPR-Cas systems are categorized into two classes, six types and over thirty subtypes that could harbor a unique (signature) type gene, and differ in the identity of the associated *cas* genes, mainly those encoding the interference module (Koonin et al., 2017; Shmakov et al., 2017b; Makarova et al., 2020a).

Some CRISPR-Cas systems have been extensively characterized, primarily due to their biotechnological relevance, which has also encouraged the search for new variants in nature (Burstein et al., 2017). However, in the existing databases, some environments are more represented than others, such as human clinical samples versus environmental samples. Among the latter, hot springs are significant for studying CRISPR-Cas because these molecular systems are widespread in the indigenous microorganisms, whether thermophiles or hyperthermophiles (Anderson et al., 2011; Weinberger et al., 2012; Weissman et al., 2019). It has been suggested that temperature impacts the viral diversity and density in these environments by decreasing mutation rates, thereby influencing the occurrence of CRISPR-Cas systems in the host microorganisms (Weinberger et al., 2012; Iranzo et al., 2013; Childs et al., 2014; Westra et al., 2016). Lower mutation rates in thermal environments are explained by the deleterious effect of substitutions at high temperatures (Drake, 2009) which would define a less diverse community than in mesophilic environments. In thermal environments, the lower virus-prokaryote ratio and lower viral community diversity (Parmar et al., 2018) translate into a lower metabolic cost for the maintenance of the CRISPR-Cas systems against viral infection compared to mesophilic environments (Westra et al., 2016). In this last environment, the spacer catalog has to adapt to more diverse invading nucleic acids (Weinberger et al., 2012; Iranzo et al., 2013; Vale et al., 2015; Westra et al., 2015; Burstein et al., 2016; Van Houte et al., 2016; Broniewski et al., 2020; Meaden et al., 2021). Beyond the high presence of CRISPR-Cas systems in thermophiles, these environments exhibit low microbial complexity compared to mesophilic environments, with fewer microorganisms harboring CRISPR-Cas systems (Burstein et al., 2016; Weissman et al., 2019; Makarova et al., 2020a).

A genetic marker for all CRISPR-Cas systems cannot be established (Koonin and Makarova, 2019; Makarova et al., 2020a). However, the Cas1 protein is the most widespread and evolutionarily conserved *cas* gene (Makarova et al., 2015, 2020a; Koonin and Makarova, 2019) and is essential for CRISPR-Cas adaptive immunity (Krupovic et al., 2014; Amitai and Sorek, 2016; Jackson et al., 2017). Therefore, *cas1* has been used to study the ecology of CRISPR-Cas (Wu et al., 2020). Notably, a protein family composed of Cas1 homologs, called casposases, which is related to the transposition of the carrier mobile genetic element (casposon) has been identified (Krupovic et al., 2014, 2016). The Casposon superfamily has been proposed for the emergence of CRISPR-Cas systems, with their terminal inverted repeats (TIRs) and casposases being the presumed ancestors of CRISPR and CRISPR-associated Cas1, respectively (Koonin and Krupovic, 2015; Krupovic and Koonin, 2016; Mohanraju et al., 2016).

The diversity of Cas1 homologs discovered in new taxa and recently explored environments suggests functions other than those described for immunity (Makarova et al., 2020b), which encourages its study in the natural environment. Despite the high prevalence of CRISPR-Cas systems in hyper/thermophiles, the phylogenomics of the Cas1 protein has not been extensively explored in thermal environments of circumneutral pH. Therefore, to deepen our understanding of the relevance of CRISPR-Cas systems at the community level, the goal of this study was to describe the phylogenetic and environmental diversity of the Cas1 protein in 20 globally distributed hot springs. We hypothesized that these environments harbor new groups of Cas1 homologs not described to date. This study recovered 2,150 Cas1 sequences using 48 metagenomes from 20 hot springs ranging from 42 to 80°C and pH 6 to 9. Our results revealed a correlation between the hot spring dissimilarity observed at Cas1 and taxonomy (16S rRNA), with geographical location as the main explanatory variable of these dissimilarities. Furthermore, several Cas1 homologs did not cluster with reference Cas1 proteins from previously described CRISPR-Cas systems but were positioned at the root of specific phylogenetic groups in the tree. Finally, some Cas1 from hot springs formed a new family of casposases (proposed family 5).

## Materials and methods

### Study sites, El Tatio sampling, DNA extraction, and sequencing

**FIGURE 1:**
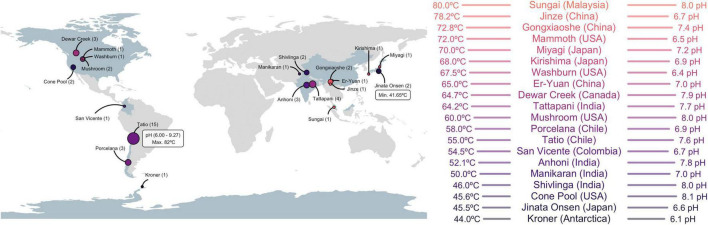
Geographic locations of hot springs used in this study **(left)**, with the number of metagenomic data sets in parenthesis (proportional to circle size). The temperature and pH limits of the survey (white box) and the average temperature and pH of each hot spring site **(right)** are indicated. Detailed metadata of each sample are provided in Supplementary Table 1.

In this study, we defined a thermophilic temperature range between 40 and 80°C and an approximately neutral pH (6–9) as the most relevant physicochemical parameters to retain hot spring samples (Meyer-Dombard et al., 2005; Inskeep et al., 2010; López-López et al., 2013; Supplementary Figure 1). Parameters excluded most acidophilic prokaryotes and hyperthermophilic archaea, for which characterization of CRISPR-Cas systems has been previously described (Andersson and Banfield, 2008; Tyson and Banfield, 2008). In total, we analyzed 48 metagenomes, 35 of which were from publicly available data representing sites in North America, South America, and Asia, while 13 were obtained in this study from microbial mats within the El Tatio geyser field, Chile (Figure 1 and Supplementary Figure 2). Some El Tatio metagenomes slightly exceeded defined limits (82°C, pH 9.27, Supplementary Figure 1), but were retained since their 16S rRNA profiles were similar to other samples from the same location (Supplementary Figure 3). The geographic coordinates, physicochemical parameters, DNA source, and accession number of all samples used in this study are available in Supplementary Table 1.

The 13 El Tatio hot springs were selected to cover the entire geothermal field (Supplementary Figure 2). Temperatures of the mat samples were measured with a forward-looking infrared camera (Fluke TiS45, WA, USA) and were corroborated on the mat with a multiparameter instrument (WTW multi 340i, NY, USA). Triplicate samples of approximately 2 ml were collected with a punch from each microbial mat, kept in cryogenic vials containing RNAlater (Thermo Fisher Scientific, Vilnius, Lithuania), and then stored at −80°C until DNA extraction.

DNA extractions of the 13 El Tatio samples were performed according to Alcorta et al. (2018). An equimolar amount of DNA (400 ng) from each replicate was pooled and sent in DNAstable tubes (Biomatrica, San Diego, CA, USA) to the Roy J. Carver Biotechnology Center (University of Illinois at Urbana-Champaign, IL, USA), where libraries were prepared using KAPA HyperPrep (Kapa Biosystems, Roche, Basel, Switzerland) and then sequenced on the Illumina NovaSeq 6000 platform (S1 flowcell, 2 × 150 bp). Quality filtering of reads for the El Tatio samples was performed according to Guajardo-Leiva et al. (2018).

### Metagenome assembly and Cas1 recovery

*De novo* assembly was performed for all 48 metagenomes using SPAdes v3.10.1 (-meta) (Bankevich et al., 2012) except for ERR372908, where MEGAHIT v1.2.9 software (–presets metasensitive) (Li et al., 2015) was used due to memory limitations. Assembly data statistics for all samples used in this study are available in Supplementary Table 1. The search for Cas1 orthologs across 48 metagenomes was done with the hmmsearch tool of the HMMER v3.3 package (Eddy, 2011), after ORF prediction with Prodigal v2.6.3 (-p meta) (Hyatt et al., 2010), using the eight updated Cas1 hidden Markov models published by Wu et al. (2020). The *E*-value cut-off (0.01) was set after standardization with one representative sample metagenome (T60, BioSample SAMN15500206 from BioProject PRJNA645256), for which recovered Cas1 candidates were thoroughly curated as described in Moya-Beltrán et al. (2019). This search yielded 3,556 candidate protein sequences. Putative Cas1 orthologs were compared against acknowledged Cas1 families present in the Conserved Domain Database v.3.16 (CDD; Marchler-Bauer et al., 2017) using CD-search (Marchler-Bauer and Bryant, 2004), hhsearch (Fidler et al., 2016), and RPS-BLAST v2.2.26 (Marchler-Bauer et al., 2002) and were retained if recognized as Cas1 by at least one of the three comparison tools with an *E*-value lower than 0.003 and 0.01 for CD-search and RPS-BLAST, respectively, and a probability higher than 81.6 for hhsearch. Next, a size filter was applied to avoid including possible chimeric proteins and limit the survey to CRISPR-Cas canonical Cas1 (Cas1 representatives smaller than 400 aa (Silas et al., 2017; Wu et al., 2020) were recovered). The 3,414 sequences fulfilling this criterium were then filtered by the relative read abundance in each metagenome. Downstream analyses did not consider sequences with an abundance below 0.1% in each sample. The 2,155 recovered Cas1 sequences were filtered for sequence redundancy at 100% aminoacidic sequence identity to remove sequences absent from public databases, considered here as possibly chimerical. The final data set for this study consisted of 2,150 Cas1 sequences.

### Taxonomic assignment of Cas1 proteins

**FIGURE 2:**
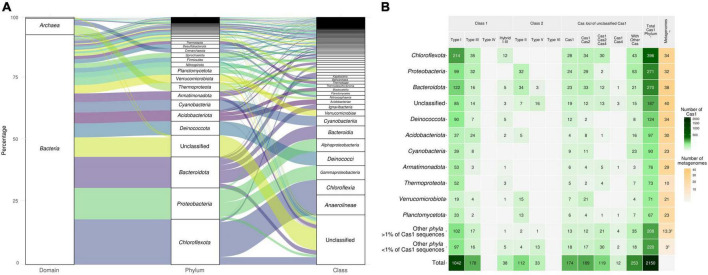
**(A)** Taxonomic distribution at the domain, phylum, and class level of Cas1 proteins from 48 globally distributed metagenome data sets (sequences over 1% are represented). Detailed taxonomic affiliation, metadata, and the aminoacidic sequence of each Cas1 protein used here can be retrieved in Supplementary Table 3. **(B)** Typification of the 2,150 Cas1 used in this study according to CRISPR-Cas system class and type. ^1^Number of Cas1 proteins at the phylum level, including the unclassified category. ^2^Number of different metagenome samples that harbor members of each phylum. ^3^Average of samples where those phyla are present.

We applied three strategies to assign taxonomy to the 2,150 Cas1 sequences recovered in this study. First, we obtained metagenome-assembled genomes (MAGs) of the 48 metagenome data sets according to Alcorta et al. (2020). Taxonomic affiliation of the MAGs was then retrieved using GTDB-tk v0.3.2 software (Chaumeil et al., 2020) with database version R89, identifying *cas1* sequences in contigs housed in the taxonomically identified MAGs. Second, for *cas1* genes not assigned through MAGs, we used the strategy of Wu et al. (2020) for Cas1 assignment and performed a BLASTp sequence similarity search against the NCBI nr database. Briefly, the five best hits were sorted according to bit score, with the best hit used for assignment if all hits belonged to the same phylum or only if the identity (%) of the best hit was at least 3% higher than the second hit (Wu et al., 2020). Finally, we checked for consistency in the taxonomic affiliation of the Cas1 sequences by retrieving the taxonomic affiliation of the non-Cas gene found in the vicinity of *cas1* in annotated metagenomic contigs (Supplementary Table 2). The assigned taxonomic affiliation of Cas1 sequences is shown in Figure 2A. Aminoacidic sequences and metadata for each sequence are available in Supplementary Table 3.

### Beta-diversity of 16S rRNA and *cas1* genes

For the final set of 2,150 *cas1* genes, reads *per* Kb *per* Gb (RPKG) were calculated for each sample using Bowtie2 (Langmead and Salzberg, 2012). Coordinate diversity analyses for the Bray–Curtis dissimilarity index were plotted using non-metric multidimensional scaling (nMDS) in the R package ampvis2 (Andersen et al., 2018). To identify sources of variation considering temperature, pH, altitude, and location (Universal Transverse Mercator, UTM, coordinates), permuted multivariate analysis of variance (PERMANOVA) (Anderson, 2001) was performed with the R package vegan (adonis2, not-sequentially added terms) (Oksanen et al., 2020). The same analyses were performed on the 2,980 16S rRNA genes (over 0.1% RPKG) obtained with MATAM software (Pericard et al., 2018). Finally, the Mantel test was used to statistically compare the 16S rRNA and Cas1 gene Bray–Curtis matrices. The sequences and taxonomic affiliation of the 16S rRNA genes, as determined with the SILVA 138 SSU database (Quast et al., 2013), are listed in Supplementary Table 4, while rarefaction curves are shown in Supplementary Figure 4.

### Cas1 subtypes and phylogenetic analysis

Phylogenetic analyses (ML) were performed with IQtree software (v.1.6.8) (Nguyen et al., 2015) [-m TEST: LG + F + G4 Le and Gascuel model (Le and Gascuel, 2008)] using ultrafast bootstrap (-bb 10,000) (Hoang et al., 2018) after Clustal Omega alignment (Madeira et al., 2019). We followed the recommendation of Wu et al. (2020) to identify Cas1 subtypes using 93 Cas1 reference sequences in the phylogenetic analyses. Additionally, we included casposase genes due to their importance in *cas1* gene evolution (Makarova et al., 2013; Krupovic et al., 2014; Krupovic and Koonin, 2016). The phylogenetic tree was displayed using the iTOL web server (Letunic and Bork, 2019) with the *Streptomyces coelicolor* transposase gene (NP_626990) as an outgroup to root the tree (Krupovic et al., 2016; Wu et al., 2020). Furthermore, the classification of Cas1 CRISPR-Cas subtypes was analyzed in parallel for each Cas1 using CRISPRCasTyper software (Russel et al., 2020), using mandatory and accessory cas-numerical-score guide typification of the tool. We decided to keep the nomenclature system of CRISPRCasTyper, where complex operons are considered hybrid (six *cas* genes from two or more types with a score of at least six and at least one specific *cas*), and not typified operons are labeled as ambiguous (non-hybrid operons with two or more *cas* subtypes and the same scoring) or false (neither hybrid nor ambiguous) (Russel et al., 2020).

### Cas1 protein similarity network and gene neighborhood analysis

The Cas1 similarity network analysis was elaborated as described by Cardenas et al. (2016). Briefly, the set of 2,150 Cas1 sequences was clustered using CD-HIT software (Fu et al., 2012) with the parameters outlined for Cas1 clustering (Makarova et al., 2011) (i.e., 90% identity over 75% coverage). The resulting 1,468 representative Cas1 genes were analyzed with BLASTp-all-against-all (default parameters) (Altschul et al., 1990) using an *E*-value of 10^–^35. Finally, the pairwise bit score was used as the distance for network visualization in Cytoscape 3.9.1 (Shannon et al., 2003) using the *organic* layout. The set of reported contig sequences containing previously identified *cas1* was used to analyze the neighborhood (Moya-Beltrán et al., 2021). Briefly, up to 10 ORFs upstream and/or downstream of *cas1* were recovered and their annotations were retrieved using a GFF file. It should be noted that differences in sequencing quality between the metagenomes used in this study (Supplementary Table 1 and Supplementary Figure 4) might have affected assembly and therefore the *cas1* genes and vicinity that could be recovered in some samples. Gene products were clustered at a similarity threshold of 0.5 and coverage threshold of 0.33 to obtain representative sequences using MMseqs2 (Steinegger and Söding, 2017). Putative functional assignment of protein clusters was done as described in Moya-Beltrán et al. (2019). Results are summarized in Supplementary Table 2.

### Putative casposase analyses

The 174 *cas1* sequences without other *cas*-encoding genes in the vicinity (±10 ORFs, Figure 2B) were deemed as putative casposase genes. According to described casposons (Krupovic et al., 2014, 2017), we decided to include *cas1* genes with at least seven genes in the contig and *cas1* not situated at the end of the contig. Seven *cas1* sequences fulfilled this criterion, all of which are allocated with casposase references in the phylogenetic analyses (Figure 4); thus, we also included the remaining three Cas1 sequences of the casposase reference clade (Figure 4) that present vicinity (Cas1_1015 was located in the casposase reference clade but without ORFs in the vicinity). In order to determine the casposase family affiliation of these sequences, we reproduced the phylogenetic analyses of Krupovic et al. (2014, 2016). EasyFig v.2.1 (Sullivan et al., 2011) was used to compare contigs of candidate sequences, for which TIRs were searched using TirVish (Gremme et al., 2013). Available TIRs belonging to described casposons (Krupovic et al., 2014) were also used as queries for identification of TIRs in putative casposase contigs of this study, including the reverse-complement strands.

**FIGURE 3:**
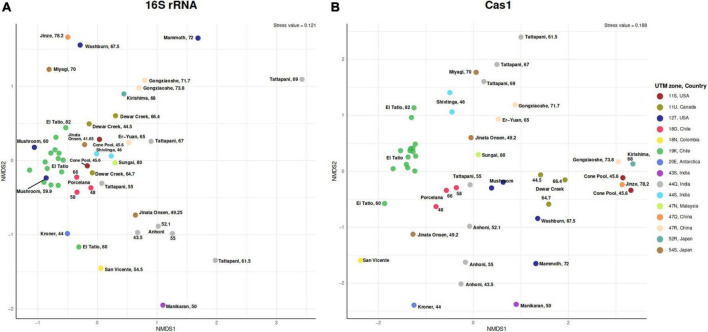
Non-metric multidimensional scaling of beta diversity (Bray–Curtis index) showing dissimilarity of the **(A)** 16S rRNA gene and **(B)** Cas1 genes from 48 metagenomes. Points are colored according to Universal Transverse Mercator coordinates (UTM) and country (right legend).

**FIGURE 4:**
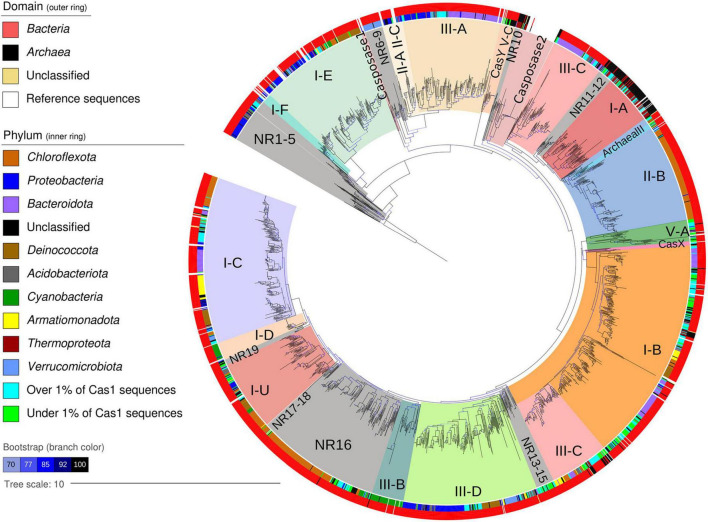
Maximum-likelihood phylogenetic tree of 2,150 Cas1 proteins from 48 global hot spring metagenomes. Domain and phylum are indicated as outer and inner rings, respectively, according to the left legend. The remaining taxa over and under 1% of Cas1 sequences are represented by one respective color. Tree clades are colored according to reference Cas1 sequences of a CRISPR-Cas system subtype mentioned by Wu et al. (2020) or casposase genes used in the phylogenetic analyses. Hot spring tree clades without reference are labeled as NR (no reference). Branch color indicates ultrafast bootstrap values (10,000 repetitions) as a percentage, over 70% in all cases. The tree was rooted using the *Streptomyces coelicolor* transposase gene (NP_626990).

## Results

### Hot spring sample characterization

Forty-eight metagenomic data sets from 20 hot springs of circumneutral pH, with temperatures in the mesothermophilic to thermophilic range, were recovered from public databases or generated herein (Supplementary Table 1) to search for global and local patterns of Cas1 diversity in thermophilic environments. The 48 metagenomic data sets represent nine countries in America and Asia (Figure 1). The El Tatio samples submitted here (15 metagenomic data sets), along with those from northern Patagonia (Porcelana, Chile; 3 metagenomic data sets) and Antarctica (Kroner; 1 metagenome), are the only ones from the southern hemisphere within the target physicochemical range (Figure 1). El Tatio sampling encompassed the 10 km^2^ area of the high altitude (∼4,200 MAMSL) geothermal field (upper, middle, and lower geyser basin, Supplementary Figure 2) in the Atacama Desert (Chile) (Glennon and Pfaff, 2003). The 48 samples comprise 36 different temperatures ranging from 41.65°C (Jinata Onsen, Japan) to 82°C (El Tatio, Chile), with an average of 57.9°C and a mode and median of 55°C. An average pH of 7.44 with a mode of 8 and a median of 7.51 was observed. The El Tatio geothermal field recorded the highest (pH 9.72; Pto13) and lowest (pH 6; T82) pH (Supplementary Table 1). Over 58% of the samples were recovered from microbial mats, while 20% were from sediments and 20% were from water fractions. No information was found on the DNA source for two samples (San Vicente hot spring from Colombia and Mammoth, Liberty Cap Streamers, USA) (Supplementary Table 1). Extending available hot spring metagenomes to the southern hemisphere balances database samples and brings new taxonomic variants for thermophile microorganisms surveys.

### Frequent hot spring taxa-affiliated Cas1 abound in thermophilic metagenomes

Of the 2,150 candidates Cas1 finally recovered from 48 metagenomic data sets, 912 (42.4%) were mapped to MAGs (654 MAGs). The remaining 1,238 (57.5%) Cas1 were taxonomically classified *via* BLASTp against the NCBI nr database (Supplementary Table 3). Approximately 92% of the assignments belonged to the *Bacteria* domain, and 7% to *Archaea*, and were affiliated with 52 phyla and 97 classes. The phylum Chloroflexota accounted for approximately 18% of the total recovered Cas1, represented mainly by classes Chloroflexia (≈8%) and Anaerolineae (≈6%) (Figure 2A). The phylum Proteobacteria (≈13%) was mainly represented by Alphaproteobacteria and Gammaproteobacteria*-*affiliated Cas1 sequences. In contrast, the Bacteroidota phylum (≈13%) pertained to three main classes (Bacteroidia, Ignavibacteria, and Kapabacteria) (Figure 2A). Thirty-five phyla and 76 classes were found below the 1% of Cas1 frequency (Supplementary Table 3). The prevalence of Cas1 in reportedly abundant bacterial hot spring taxa corroborates the importance of CRISPR-Cas systems in hot springs and points out these environments as models to study the environmental microbiology of CRISPR-Cas and their alternative functions (Mohanraju et al., 2022).

### High frequency of class 1-type I Cas1 proteins in hot springs

Consolidated typification using phylogeny, CRISPRCasTyper, and vicinity approaches (Supplementary Table 3) allowed us to classify most Cas1 (1,403 of 2,150, 65.2%) to a CRISPR-Cas type/subtype. An overall predominance of class 1-type I (1,042, 48.4%) was observed (Figure 2B), whereas class 1-type III was the second most represented type, followed by class 2-type II (Figure 2B). Thirty-eight Cas1 sequences were classified in type I-type III hybrid operons. No class 1-type IV and class 2-type VI systems were found. For the 174 (8%) Cas1 without *cas* in the neighborhood (Figure 2B), further analysis was performed to elucidate their relevance as Cas1 not belonging to a *bona fide* CRISPR-Cas locus, as casposase proteins or *cas1*-solo (non-casposase *cas1* without *cas* genes in the neighborhood) (see below). The Cas1 type frequencies observed in hot springs corroborate the predominance of class 1 CRISPR-Cas systems, suggesting that temperature could not be the main driver of CRISPR system types in nature.

### Geographical location as the main driver of *cas1* variance

16S rRNA and *cas1* gene occurrence and abundance *per* metagenome were used to calculate the Bray–Curtis dissimilarity indexes. At the 16S rRNA gene level (Figure 3A), hot springs belonging to the same UTM zone were more similar than those from different UTM zones. However, samples of intermediate temperature (>55°C < 68°C) from different UTM zones were more similar than samples at the upper and lower end of the temperature range (Figure 3A). This may indicate the existence of a higher gene flow between hot springs in the middle-temperature range (55–68°C), regardless of their geographic origin. In contrast, temperatures at the upper and lower extremes could restrict gene flow even between nearby geographic zones. Although no significant diversity differences were observed between the *cas1* and 16S rRNA according to the positive correlation (0.57, 0.0001) found by the Mantel test (Supplementary Table 5), the *cas1* data (Figure 3B) show that samples were geographically more structured by UTM zone than the results observed for 16S rRNA. The PERMANOVA of Bray–Curtis distances indicates that geographical location explained 47 and 42% of the observed variance in the Cas1 and 16S rRNA, respectively (Supplementary Table 5). This result is consistent with the nMDS, where the influence of geographic location is more evident at the Cas1 level than at the 16S rRNA level. Other variables, such as temperature, pH, or altitude, scarcely contributed to the variance. Even, a higher sequential effect of geographical location was observed when the sources of variation were added according to the marginal PERMANOVA (Supplementary Table 5). All these results support the existence of dispersion barriers in hot springs.

To corroborate the relevance of geographical location, we looked for environmental variables affecting the genetic diversity of Cas1. We analyzed Cas1 at the sequence level using a genetic distance matrix (percent identity). The marginal PERMANOVA with distance matrix values revealed that all sources of variation considered here poorly explained the differences at the sequence level (Supplementary Table 5), suggesting that Cas1 critical structure is very conserved in hot springs, at least in the canonical protein size used here.

### Novel subclades of hot spring Cas1

A rooted phylogenetic tree was constructed with the 2,150 sequences of Cas1 proteins from the 48 globally distributed hot springs (Figure 4), along with 93 Cas1 reference sequences that helped typification (Wu et al., 2020). Most hot spring Cas1 sequences clustered with the reference sequences, showing that hot spring Cas1 are linked to CRISPR-Cas systems of diverse known subtypes (Figure 4). However, 19 clades without reference sequences were also obtained, harboring poorly classified or unclassified Cas1 sequences (labeled NR, i.e., No Reference). Except for subclade NR16, NR clades were usually positioned next to the root of the tree or the root of internal reference Cas1 clades (Figure 4, NR marked in gray). NR clades and their position indicate that some Cas1 from hot springs are infrequent in databases or completely new, and also suggest that Cas1 sequences close to casposase clades may be related to casposons.

**FIGURE 5:**
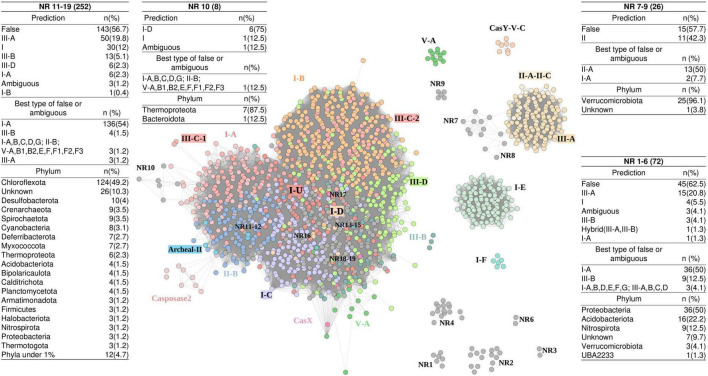
Similarity network of 1,468 representative Cas1 proteins obtained from 2,150 Cas1 from 48 hot spring metagenomes. Points are colored according to the clades of the phylogenetic tree in Figure 4. Specific consolidated typification of NR (no reference) Cas1 clades is indicated in boxes, where the nomenclature of “ambiguous” or “false” was taken from Russel et al. (2020), meaning non-hybrid operons with two or more *cas* subtypes and the same scoring, or neither hybrid nor ambiguous operons, respectively. NR5 is represented by one sequence and as a singleton in the network (not shown).

Overlayed taxonomic affiliation showed that 52 microbial phyla were represented at least once in the tree (Figure 4, outer and inner rings). Cas1 from the same bacterial phyla frequently clustered together, and below the phylum level, no clear associations were recovered (data not shown). However, Cas1 proteins from predominant phyla such as *Chloroflexota* (396 sequences) and *Proteobacteria* (271 sequences) were distributed in several clades within the tree (Figure 4). Most of the 90 Cas1 sequences from members of the phylum *Cyanobacteria* were clustered into clades recognized as I-D and III-B (Figure 4). Metadata overlay on the tree (e.g., temperature) was of poor value for revealing specific data patterns (Supplementary Figure 5), except for Cas1 clades I-A and I-B belonging to *Archaea*, which were associated with slightly warmer temperatures. Regarding the taxonomy of NR subclades (358 total sequences), most belonged to *Chloroflexota* (124, 35%) and *Proteobacteria* (39, 11%), whereas taxonomically unassigned (phylum-level) Cas1 sequences ranked third (34, 9.5%), which could suggest new Cas1 homologs, especially for those deep clades. In general, phyla that harbored most of the Cas1 sequences (Figure 2) also harbored most of the NR members (over 2%, Figure 5), indicating that predominant hot spring taxa could harbor rare Cas1 homologs.

To further explore the nature and characteristics of the Cas1 NR subclades, we constructed a similarity network using the 1,468 representative Cas1 proteins of the aforementioned phylogenetic tree (Figure 4). Each node of the network (Figure 5) represents a Cas1 protein, and edges correspond to the bit score of an all-versus-all BLASTp analysis. The largest network module contains three principal regions conforming a core, where (1) most I-A, II-B, III-C-1, and Archaeal-II Cas1 sequences are separated from the region composed of (2) I-C, and (3) I-B, III-C-2, and III-D subtype proteins. Some nodes are separated by long edges and arranged as “satellites”: Casposase2, CasX, and some Cas1 of subclade V-A (Figure 5), which indicates unusual Cas1 homologs. The second-largest module harbors Cas1 of subtypes II-A-II-C and III-A, together with “satellite” sequences (NR7 and NR8, Figure 5). The third module is composed exclusively of I-E sequences, which in Figure 4 also clustered apart with I-F representatives (a minor yet separate module in the network; Figure 5). Finally, Cas1 of the rare subtype CasY formed an isolated module. Several small NR modules are isolated from the rest (NR1–NR6). However, the biggest NR clade of Figure 4 (NR16) was inside the main network module (Figure 5), suggesting new varieties of Cas1 similar to traditional Cas1 sequences.

Most NR Cas1 clades of the phylogenetic tree were classified as false or ambiguous (210, 9.7%), but also several sequences were affiliated with a CRISPR-Cas system type/subtype (Figure 5 and Supplementary Table 3). According to the network arrangement, the main module harbors 252 sequences of subclades NR11–NR19, where 146 (58%) are classified as false or ambiguous, and 106 (42%) effectively belong to a known CRISPR-Cas system (Figure 5 and Supplementary Table 6). Interestingly, modules NR7, NR8, and NR9 only include sequences classified as type II Cas1 or false. The NR10 cluster is predominantly of type I-D Cas1 sequences and is displayed as “satellite” of the main cluster, which is also observed for NR7, NR8, and Cas1 sequences of the Casposase2 clade (Figure 5). These results show that Cas1 from hot springs harbor rare sequence variants. Deeper analyses of “satellite” casposase clade allowed us to identify new Cas1 unrelated to CRISPR-Cas immunity.

### New casposase genes from hot springs

**FIGURE 6:**
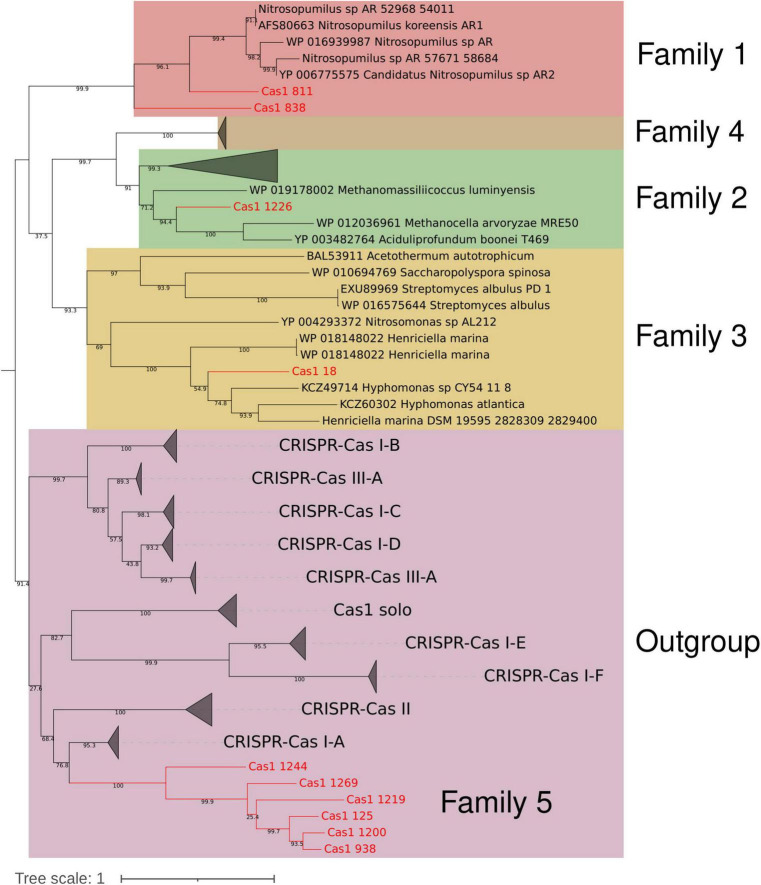
Reconstructed maximum likelihood phylogenetic tree of Krupovic et al. (2014, 2016). Ten hot spring casposases (colored red and with ID indicated in Supplementary Table 3) were analyzed along with 110 reference casposases. Clades are colored according to the described casposase family (1–4) or outgroup, except family 5, which is indicated inside the outgroup in red, close to Cas1 of subtype I-A CRISPR-Cas system. Bootstrap values are indicated in branches as a percentage.

**FIGURE 7:**
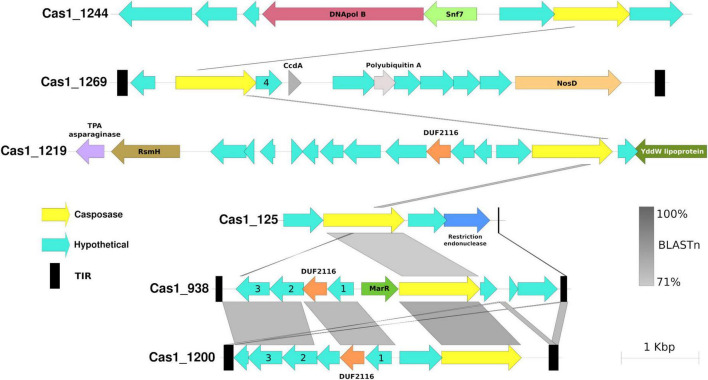
Genetic neighborhood comparison of family 5 casposase genes from hot springs. Casposase gene ID is indicated at the beginning of the contig, and the BLASTn pairwise percentage is indicated according to the color shade. The legend indicates casposases (yellow), hypothetical proteins (aqua green), and terminal inverted repeats (black). Hypothetical proteins with numbers inside refer to the same protein sequence (another hypothetical protein number 4 can be found in Supplementary Figure 6, Cas1_18 of family 3 casposases). TIRs could not be identified in Cas1_1219 and Cas1_1244.

Performing the casposase phylogenetic analyses of Krupovic et al., 2014, 2016, 6 of 10 Cas1-solo from hot springs were located in a monophyletic clade in the outgroup, close to Cas1 subtype I-A (Figure 6), representing a novel branch of casposases. We propose that this clade corresponds to a new family of casposases (family 5) discovered in hot springs. Other hot spring casposases grouped with already known families (Figure 6). New family 5 and Cas1 proteins belonging to known casposase families show great neighborhood genetic diversity (Figure 7 and Supplementary Figure 6). Identity comparison also revealed great variation, where only contigs harboring Cas1_938 (Miyagi, Japan) and Cas1_1200 (Washburn, USA) presented greater similarity (Figure 7). The same was observed for the gene content of the contig, where the most distant contig (Cas1_1244) is the only one harboring DNApol B. TIRs were identified in five contigs (Cas1_18, Cas1_938, Cas1_1200, Cas1_1226, and Cas1_1269). For sequence Cas1_1226, the TIR was identified by alignment with the TIR *Candidatus* “Acetothermum autotrophicum” (Krupovic et al., 2014). These results reveal the remarkable variation of genetic content inside family 5; however, casposases of this family show a high identity between them compared to other families (Supplementary Figure 7). The genetic neighborhood of remaining non-family 5 putative casposase gene contigs are available in Supplementary Figure 6.

## Discussion

### Expanding the *cas1* gene information to the southern hemisphere

The metagenomes used in this study included new El Tatio metagenomes from El Tatio geothermal field in Chile, expanding the metagenomic data available for hot springs from underrepresented geographical zones. In addition, this study contributed to the expansion of the known Cas1 sequence from the southern hemisphere, including Antarctica (Figure 1). The global and regional distribution of the metagenomic data used allowed us to compare the diversity of 16S rRNA and Cas1 on a continental scale, but also locally, as in the case of the El Tatio geyser field. The ranges of temperature (41–80°C) and pH (approximately 6–8) used for this study have been defined as suitable for thermophilic microorganisms (Zablocki et al., 2018; Merino et al., 2019) and reveal that metagenomic samples in the mesothermophilic range of temperature (approximately 55–65°C) are globally similar, corroborating that temperature and pH are the main drivers of microbial diversity in hot springs (Massello et al., 2020). Nevertheless, the local adaptation revealed by some Cas1 suggests the presence of dispersal barriers to gene flow which may be associated with variables not quantified in this study (Figure 3B).

The metagenomic data sets used here corroborate the presence of previously described phyla abundant in hot springs (Klatt et al., 2011, 2013; Inskeep et al., 2013; López-López et al., 2013; Bolhuis et al., 2014; Sharp et al., 2014; Strazzulli et al., 2017). Most hot spring metagenomes share a small core of highly represented taxa, such as the phylum *Chloroflexota*, and several minor phyla (Figure 2 and Supplementary Figure 3). However, the species turnover evidenced by the 16S rRNA gene Bray–Curtis dissimilarity index, was less than the turnover for the *cas1* gene (Figure 3), which was more geographically structured. This is consistent with the degree of isolation and local evolution previously suggested for thermal environments and some phyla, such as thermophilic *Cyanobacteria* (Finsinger et al., 2008; Kunin et al., 2008; Ionescu et al., 2010). There are some exceptions, such as the case of Tattapani, which showed high dissimilarity for the 16S rRNA gene, but not for *cas1* (Figure 3). One possible explanation could be a local adaptation of CRISPR-Cas systems to similar conditions and, for example, confronting a similar viral community. Meanwhile, other samples, such as from Gongxiaoshe, showed high similarity in the 16S rRNA gene but great dissimilarity with *cas1* (Figure 3). This could be due to the presence of similar bacterial communities versus locally different viral communities, which would support differential adaptation of the host adaptation module revealed by *cas1*. We hypothesize that Cas1 may expose local adaptations due to the specificity of virus-host relationships (Zablocki et al., 2018) mediated by CRISPR-Cas systems in hot springs. This hypothesis is consistent with the argued rapid evolution observed in extreme environments (Li et al., 2014) and the specificity of prokaryotic genes (Coelho et al., 2022), which could also be necessary for thermophilic viral communities. *Cas1* beta-diversity variations can also suggest that the same host could have existed at the beginning of several hot spring communities, where today viruses may help reveal evolutionary changes in these host communities.

### High *cas1* abundance in prevalent hot spring phyla

Abundances of *cas1* and 16S rRNA genes showed similarities in each sample regardless of hot spring temperature (Supplementary Figure 3), especially for predominant phyla such as *Chloroflexota, Proteobacteria, Cyanobacteria*, and *Bacteroidota*, which highlights the role of CRISPR-Cas systems. Cas1 protein diversity analyses at the community level are scarce, and few surveys consider predominant hot springs taxa and CRISPR-Cas types, particularly for *Chloroflexota* and *Bacteroidota* phyla. Wu et al. (2020) observed that *cas1* prevalence in soil samples increased with temperature in some taxa (including *Chloroflexota* and *Deltaproteobacteria*) while *rplB* abundance decreased. Differences here could be explained due to the stability of hot springs versus temperature as altering the microbiostasis of soil. However, it should be noted that the low sequencing depth of some samples used here (M46_SRR2625865, M46_SRR2626160, M61_SRR3961741, M50_ERR1543536, M4564_SRR6941191, and M65_MR4530144) could overestimate diversities found. In any case, the maintenance or removal of low-coverage samples does not alter the statistical significance of the positive correlation between 16S rRNA and *cas1* genes (Supplementary Table 5). As suggested (Weinberger et al., 2012; Iranzo et al., 2013), temperature changes could determine differential fitness for CRISPR-Cas systems (affecting the viral diversity/density of specific taxa) in the long term, which may explain the correlation observed in soil. A high prevalence of Cas1 in major taxa from hot springs can reveal a community where predominant species, mainly phototrophic, could be competition and adaptive defense specialists due to low viral diversity/density. CRISPR-Cas in predominant taxa from hot springs, especially in phototrophic species (*Chloroflexota* and *Cyanobacteria* phyla), could maintain the steady state of the system, ensuring the inflow of energy.

### The novelty of the *cas1* gene in hot springs

Reference sequence-guided typing (Wu et al., 2020) of previously described Cas1 sequences helped identify the CRISPR-Cas system category likely to be most relevant in these thermal environments. However, this approach has limitations because the *in silico* classification of CRISPR-Cas into a specific type/subtype must be based not only on phylogeny but also on the sequence similarity, genetic vicinity, domains, and catalytic residues (Makarova et al., 2020a). For this reason, we further employed CRISPRCasTyper software (Russel et al., 2020), to retrieve information in the contigs harboring *cas1* that showed type I and III CRISPR-Cas as predominant in hot springs (Figure 2), as well as in databases (Koonin et al., 2017; Crawley et al., 2018). Type I and III systems accounted for 48 and 8.2%, respectively, of the Cas1 obtained herein, which is below the total distribution of CRISPR-Cas systems reported in nature (Crawley et al., 2018). Despite type IV systems having been previously found in hyper/thermophiles (Jung et al., 2016; Taylor et al., 2019; Makarova et al., 2020a), we did not detect type IV Cas1 genes in the analyzed metagenomes. This might be due to the almost total absence described for the adaptation module in type IV CRISPR-Cas systems (Pinilla-Redondo et al., 2020), being overlooked here due to the methodological approach used. Conversely, the fact that class 1 systems are predominant in both thermophilic and mesophilic environments suggests that selective pressure is not related to temperature and mutation rates, but to specific mechanistic properties (Shmakov et al., 2017b). In this sense, class 1 systems could be more versatile in performing several functions than class 2 systems or in escaping anti-immunity mechanisms, for example, evolving just one subunit targeted by anti-CRISPR. Regarding class 2, 146 (6.7%) of Cas1 were classified as type II and V (Figure 2). Most of the Cas1 classified as type V belong to rare phyla such as *Patescibacteria* or unclassified bacteria (Figure 2), which encourages the search for new variants of this type already described in thermophiles (Chen et al., 2019; Tian et al., 2020). The absence of type VI Cas1 could be expected because these CRISPR-Cas systems target RNA (Makarova et al., 2020a), maybe pointing to scarce foreign RNA entering the cell. However, as mentioned for system IV, *cas1* genes associated with type IV systems have only been described in a few subtypes (Makarova et al., 2020a). Furthermore, described bacterial species that harbor type VI systems are mesophilic and related to humans or pets, which suggests a low prevalence of this type in hot springs.

Several Cas1 sequences could not be classified to a described system type/subtype despite being together with other *cas* genes (Figure 2). Given the genetic context, phylogeny, and network data, we speculate that some of these Cas are part of novel CRISPR-Cas systems. It should be noted that our approach was based on the tree topology, *cas* locus, and CRISPR array [using CRISPRCasTyper (Russel et al., 2020)]; however, missing contig information could hinder its assignment to a CRISPR-Cas system. Nevertheless, Cas2 and Cas4 were the most frequent *cas* gene in NR Cas1 clades. Regarding non-*cas* genes, prevalent genes were encoding hypothetical proteins as not in the CDD database (426 ORFs), RNase H-like (26 ORFs), and DUF697 or DUF370 domain-containing proteins. The fact that most gene neighborhoods are related to DNA metabolism corroborates the evidence of non-canonical functions of CRISPR-Cas systems and Cas1 (Sampson and Weiss, 2013; Krishnan et al., 2020; Mohanraju et al., 2022). Ongoing work will allow us to eventually characterize new molecular systems involving Cas proteins, which exceeds the current objectives of this work.

Our phylogenetic reconstruction is in agreement with the topology retrieved by Wu et al. (2020), but also includes several clades without reference sequences (NR), located at the root of the tree and in several internal clades (Figure 4). The phylum *Chloroflexota* harbors most of the Cas1 NR clades, which may be explained by the dominance of diverse members of this phylum in hot springs. The *Chloroflexota* Cas1 sequences were distributed in tree clades II-B, NR16, NR18, and I-U (Figure 4 and Supplementary Table 6), suggesting great diversity of adaptive immunity in this phylum. According to the data (Makarova et al., 2011, 2013, 2020a; Burstein et al., 2016), hot springs Cas1 from *Chloroflexota* includes types I and III, with a predominance of type I-A. However, in most cases, members that group in the tree with type II-B reference sequences (WP080019870 and WP011139432) belong to I-B (Supplementary Table 3), suggesting horizontal transfer of Cas1. The expected scenario was observed for C*yanobacteria*, where sequences were located as described in I-E (Makarova et al., 2013; Burstein et al., 2016), III-B (Makarova et al., 2013, 2020a; Burstein et al., 2016), and the almost phylum-exclusive I-D (Cai et al., 2013). Our results maintain the absence of type II for *Cyanobacteria* and *Chloroflexota* (Makarova et al., 2020a; Figure 4), with the majority of Cas1 of this type found in *Proteobacteria* and *Bacteroidota*, more represented by non-photosynthetic taxa (Figure 2B and Supplementary Table 3), suggesting a relationship between photosynthesis and the scarcity of the Cas9 protein. Overall, it is difficult to describe the diversity of Cas1 inside these hot spring-predominant taxa using taxonomy (despite the exclusiveness of some subtypes, e.g., I-D). As mentioned for the CRISPR-Cas system (Makarova et al., 2015, 2020a), the presence of the same type/subtype in several taxa could suggest no specific function of Cas1 in a host. Furthermore, the fact that hot springs did not reveal a new association regarding phylum-Cas1 type descriptions suggests that temperature has a minor selective effect for the CRISPR-Cas type/subtype. High horizontal gene transfer and viral infection events regulating CRISPR-Cas systems (Makarova et al., 2015; Koonin et al., 2017; Landsberger et al., 2018) could also blur the evolutionary history of Cas1 in hot springs, a hypothesis framed in the “guns for hire” model (Koonin et al., 2020). However, the case of I-D in *Cyanobacteria* points to a particular virus infecting them or specific function not necessarily related to adaptive immunity (Mohanraju et al., 2022), which could maintain Cas1 as a “not for hire” gun.

Finally, Casposase analysis highlights the novelty of Cas1 from hot springs and confirms the relevance of CRISPR-Cas in these environments. Previous work on hot springs Cas1 diversity defined four casposase gene families and a Cas1-solo outgroup, indicating that those Cas1-solo were probably vestigial genes due to non-conserved catalytic residues (Krupovic et al., 2014, 2016). With new metagenomic data sets available today, we have identified a new casposase gene group (proposed family 5, Figure 6), the closest in the tree to the Cas1-solo outgroup mentioned by Krupovic et al. (2014, 2016) and next to subtype I-A Cas1. The CRISPR-Cas subtype I-A has a majority representation in the *Archaea* domain (Makarova et al., 2015), which is observed in Cas1 subtype I-A of the tree (Figure 4), but also for family 5 casposases, suggesting a vestigial metabolic function of Cas1 related with that domain. Conversely, family 5 shows conserved catalytic site residues (Supplementary Figure 7), suggesting that they could be active enzymes. Nevertheless, casposase Cas1_1244 is the only family member with DNApol B and shows the lowest identity value with the rest of the family 5 (Supplementary Figure 7), whose remaining members share over 50% identity. Active site residues and the absence of DNAPol in most of the family 5, contrary to other Casposase families (Krupovic et al., 2014, 2017), suggest that they are functioning as non-self-replicative transposons, which is in line with the non-relationship of casposons with eukaryotic self-synthesizing transposons (Krupovic et al., 2014). We speculate that family 5 represents an intermediate stage between casposases from families 1 to 4 and Cas1 of CRISPR-Cas systems, suggesting that inactive Cas1-solo [group 1 of Krupovic et al. (2014)] and family 5 might represent recent ancestors in the evolution of Cas1. The HTH C-terminal domain identified in family 2 casposase (Hickman and Dyda, 2015) was not observed in family 5 casposase (data not shown), supporting its position as an ancestor of CRISPR-Cas Cas1. The high diversity of the genetic context of family 5 casposons, composed of poorly conserved hypothetical proteins (Figure 7), as well as their low sequence similarity concerning other families (Supplementary Figure 7), also suggest their rapid evolution, perhaps influenced by several potential horizontal gene transfer events. Only *loci* Cas1_938 and Cas1_1200 of family 5 share more than one hypothetical protein, which is intriguing because of their origin from very distant hot springs (Japan and USA, respectively, Supplementary Tables 1, 3). New casposases help to shed light on the function and evolution of hot spring casposons which will also contribute to the study of the evolution of CRISPR-Cas systems, thereby revealing potential new features that would allow for better elucidation of the origin of the system.

The present study extends the knowledge of Cas1 diversity in thermal environments, where ecological diversity was associated with local characteristics according to geographical origin. Phylogeny and network analyses reveal new Cas1 homologs, including a new family of casposons that formally extends the currently known diversity of the gene. This work could contribute to a better understanding of the evolution of CRISPR-Cas systems by describing new variants in new genetic contexts obtained from new hot springs metagenomes. This study also corroborates that hot springs are suitable environments for obtaining novel information on CRISPR-Cas ecology and evolution, and could contribute to understanding the higher prevalence of CRISPR-Cas systems in these environments.

## Data availability statement

All data generated or analyzed during this study are included in this published article and Supplementary material. Metagenomic datasets from El Tatio can be found in the NCBI database under BioProject PRJNA858297 (https://www.ncbi.nlm.nih.gov/sra/).

## Author contributions

OS, SG-L, CB, CR, and BD made the field sampling. OS, SG-L, AM-B, and JT-L made *in silico* analyses. RQ, FJMM, and BD contributed significantly to the research design and writing process. OS and BD conceived the study and wrote the manuscript. All authors contributed to the article and approved the submitted version.
