# Supplementary material for: Global phylogenomic novelty of the Cas1 gene from hot spring microbial communities
Source: Front Microbiol. 2022 Dec 2;13:1069452. doi: 10.3389/fmicb.2022.1069452 (PMC9755687; doi:10.3389/fmicb.2022.1069452)
Supplement: Supplementary file 1 [file Data_Sheet_1.PDF]

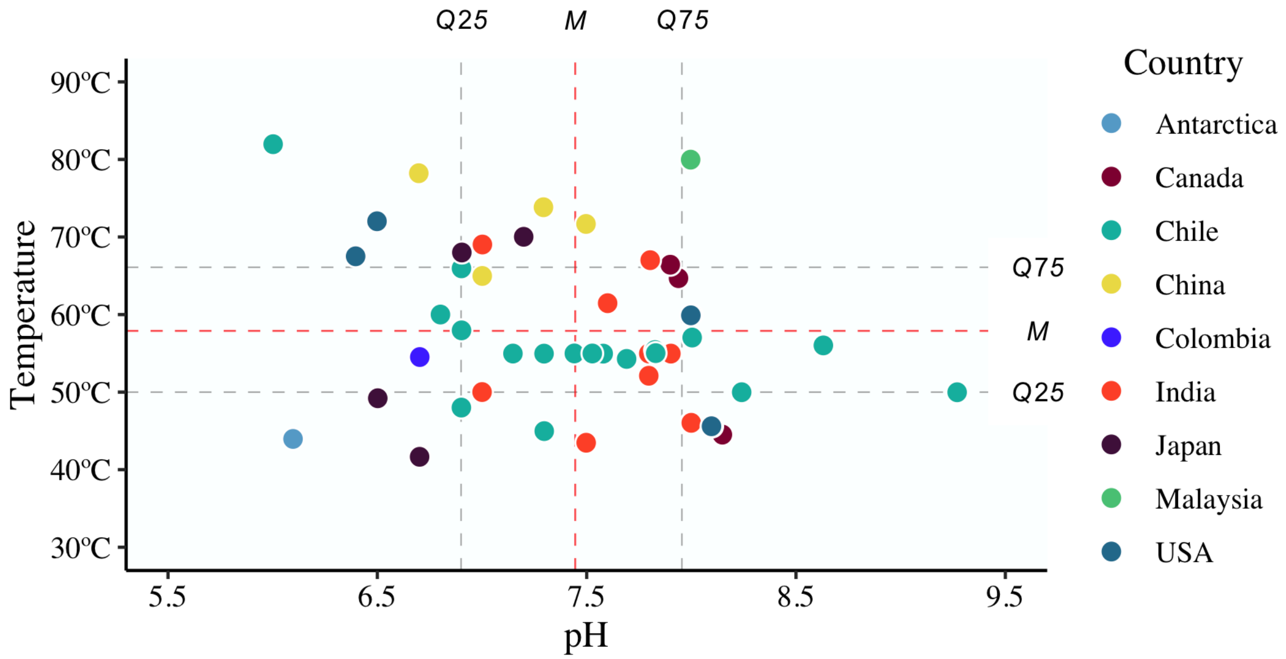

Supplementary Figure S1. Distribution of 48 hot spring metagenomes used in this study arranged according to temperature and pH. Dashed lines showing quartiles 25 and 75. Points are colored according to the countries of the right legend.

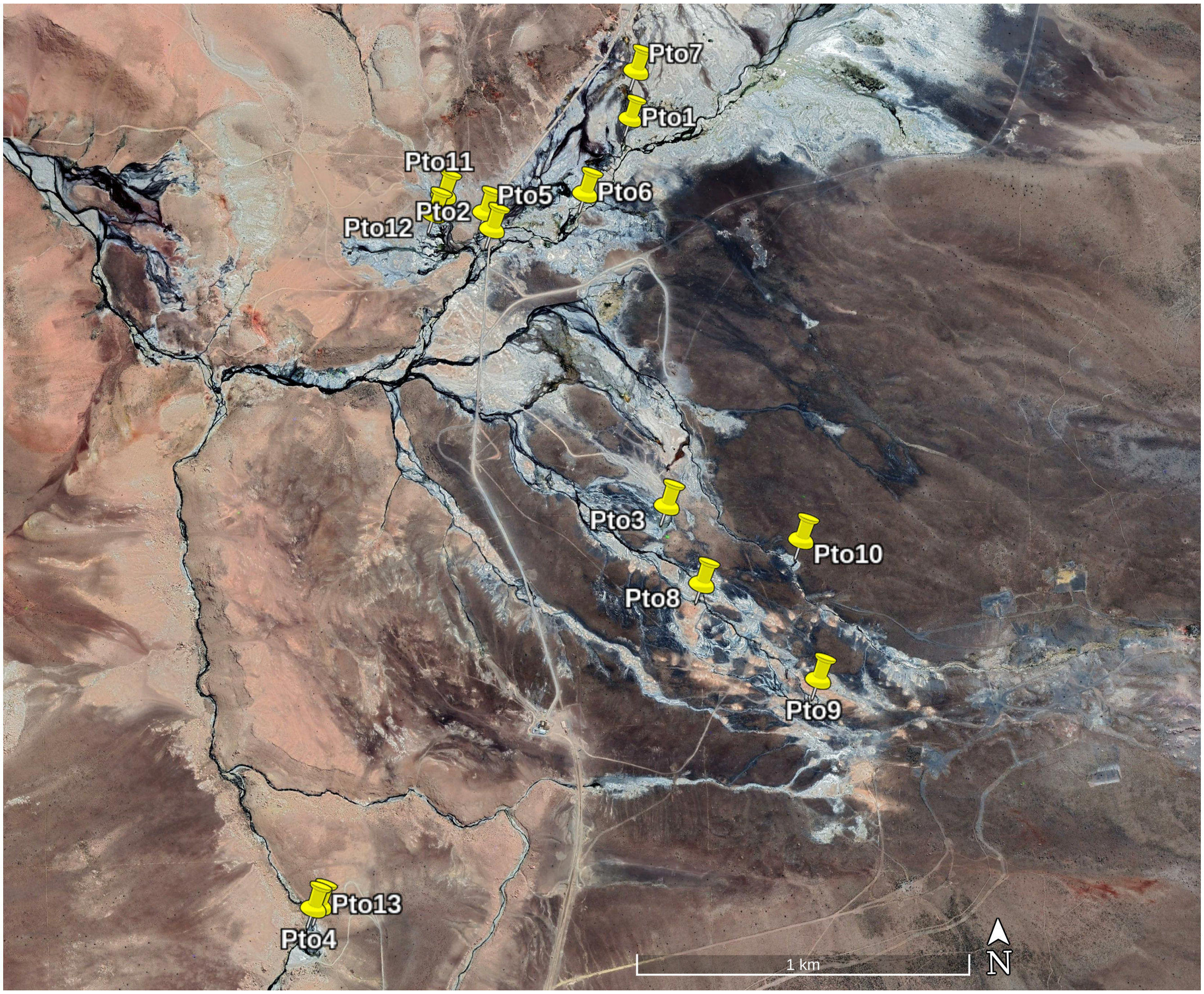

Supplementary Figure S2. Location of 13 metagenomes obtained here from El Tatio geothermal field. Additional information such as temperature, pH and geographic coordinates, of each sampling point is available in Supplementary Table S1.

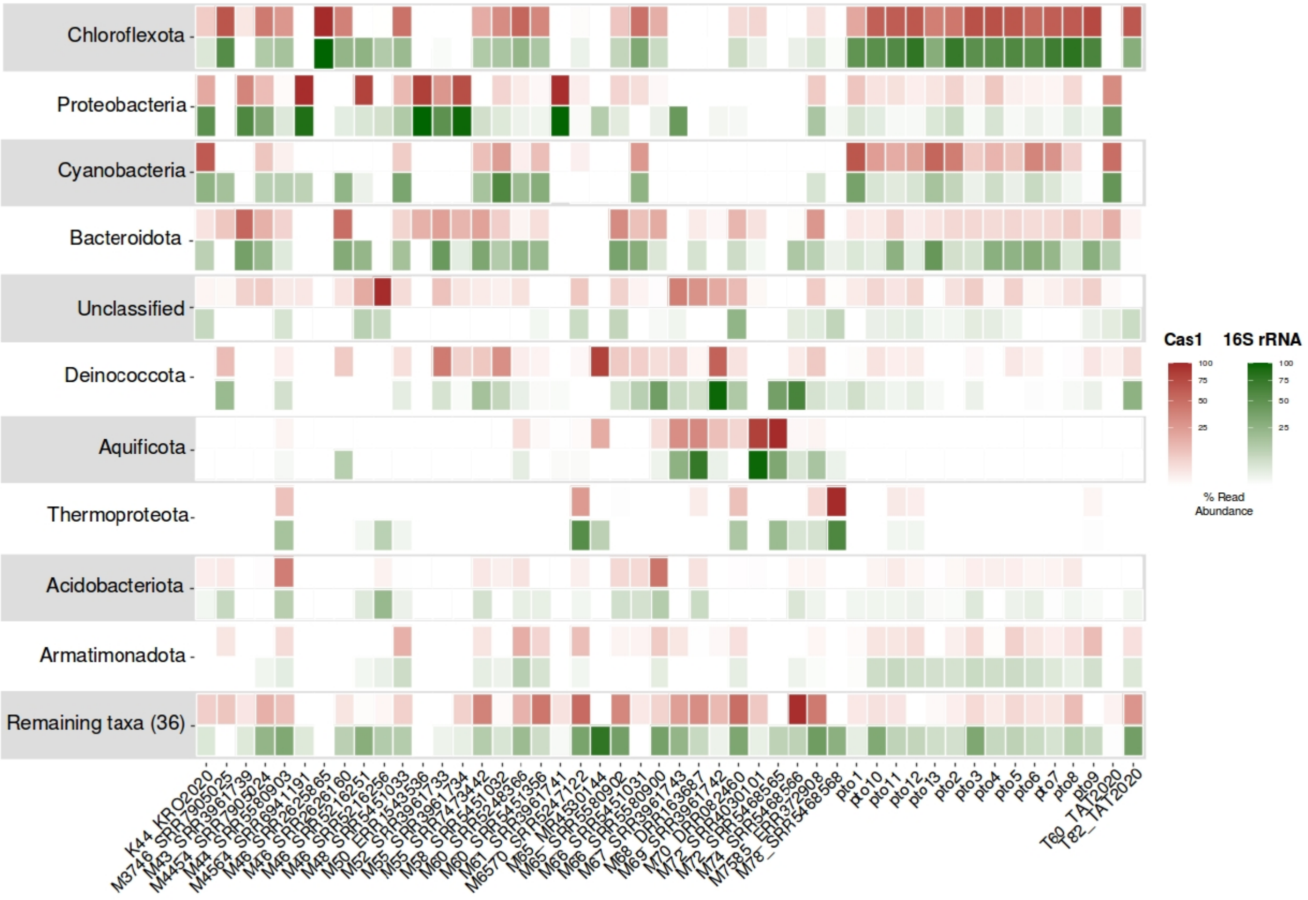

Supplementary Figure S3. Relative abundance of the 16S rRNA and *cas1* genes from the 48 metagenomes used in this study. Metagenome ID is indicated at the bottom and taxonomic affiliation at the phylum level is shown on the left. Additional information for each sequence used in this study, considering detailed taxonomic affiliation and metadata from the hot spring, is available in Supplementary Table S4 for the 16S rRNA gene, Supplementary Table S3 for the Cas1 gene and Supplementary Table S1 for the metadata.

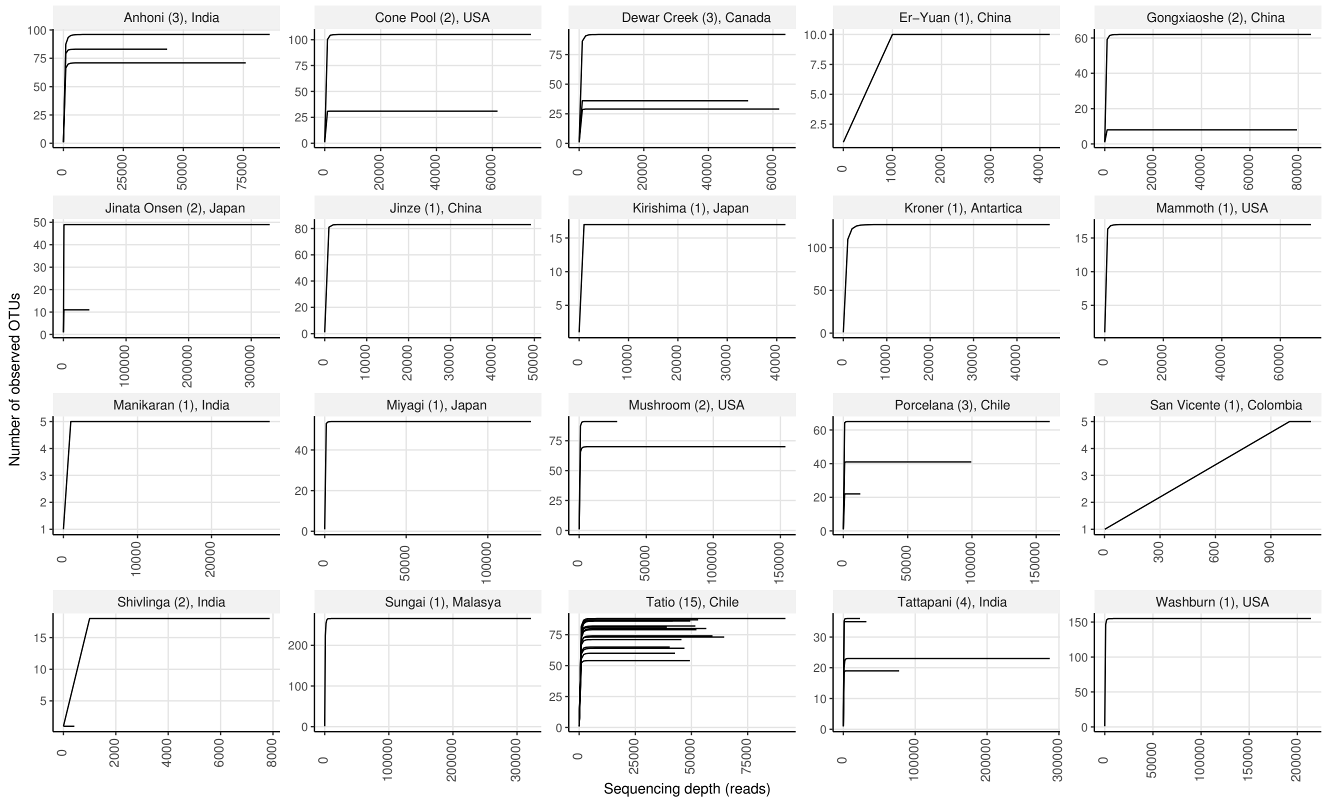

Supplementary Figure S4. Rarefaction curves of the 48 metagenomic data sets used in this study grouped according to the 20 hot spring sources. Parentheses indicate the number of metagenomes obtained in each hot spring. Additional information about the quality of the metagenomes and the assembly is available in Supplementary Table S1.

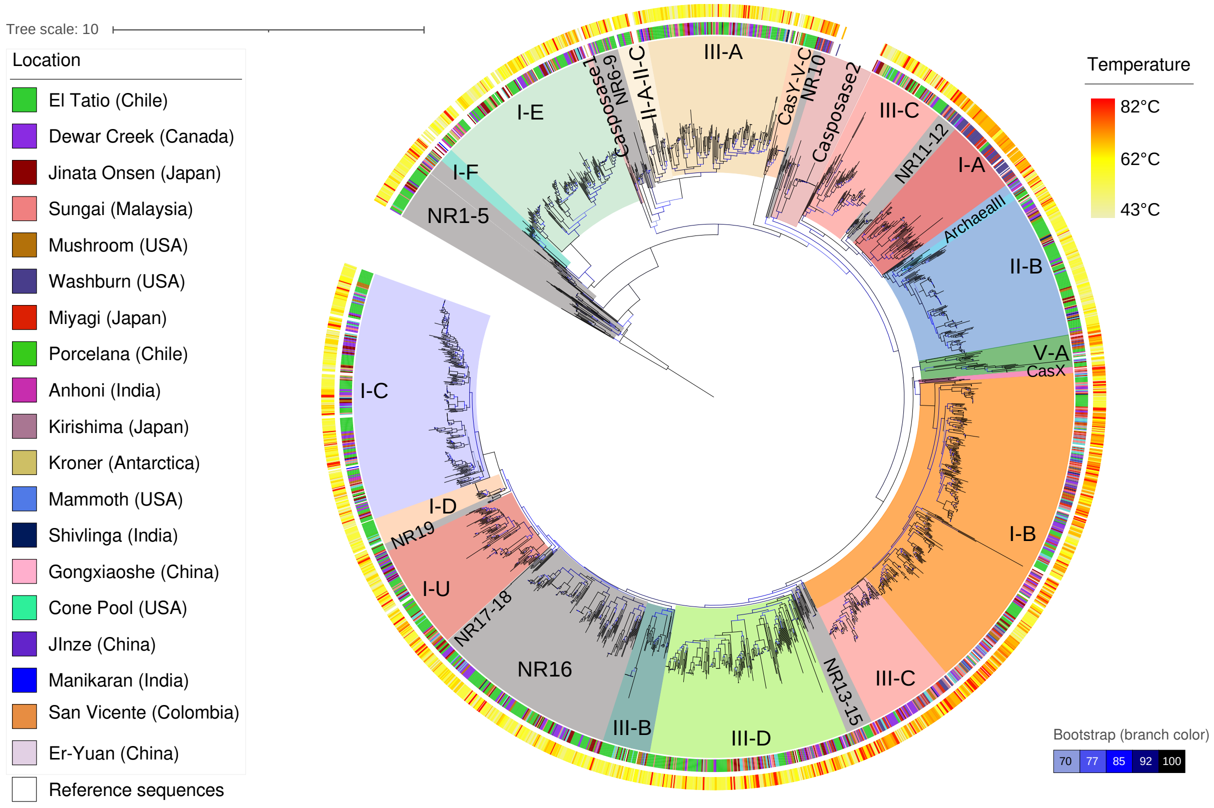

Supplementary Figure S5. Maximum-likelihood phylogenetic tree of the 2150 Cas1 proteins from 48 global hot spring metagenomes. The temperature of the hot spring and hot spring are indicated as outer and inner rings, respectively, according to the left legend. Tree clades are colored according to reference Cas1 sequences of a CRISPR-Cas system subtype described by Wu et al. [32] or casposase genes used in the phylogenetic analyses. Hot spring tree clades without reference are labeled as NR (no reference). Branch color indicates ultrafast bootstrap values (10000 repetitions) as a percentage, over 70% in all cases. The tree was rooted using the *Streptomyces coleicolor* transposase gene (NP_626990).

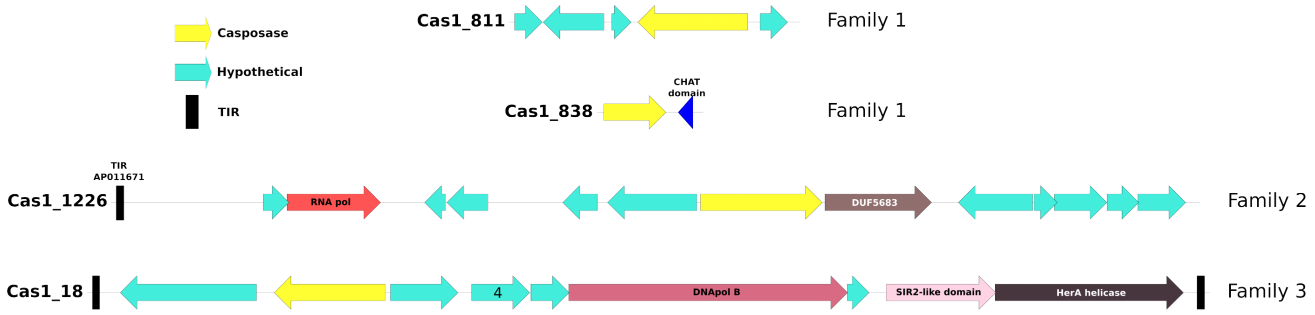

Supplementary Figure S6. Genetic neighborhood of casposase genes of families 1, 2 and 3 recovered from hot springs. Casposase gene ID is indicated at the beginning of the contig. The legend indicates casposases (yellow), hypothetical proteins (aqua green) and terminal inverted repeats (black). The hypothetical protein with number 4 inside refers to the same hypothetical protein 4 of family 5 Cas1_1269. TIRs could not be identified in Cas1_811 and Cas1_838 due to truncated contigs.

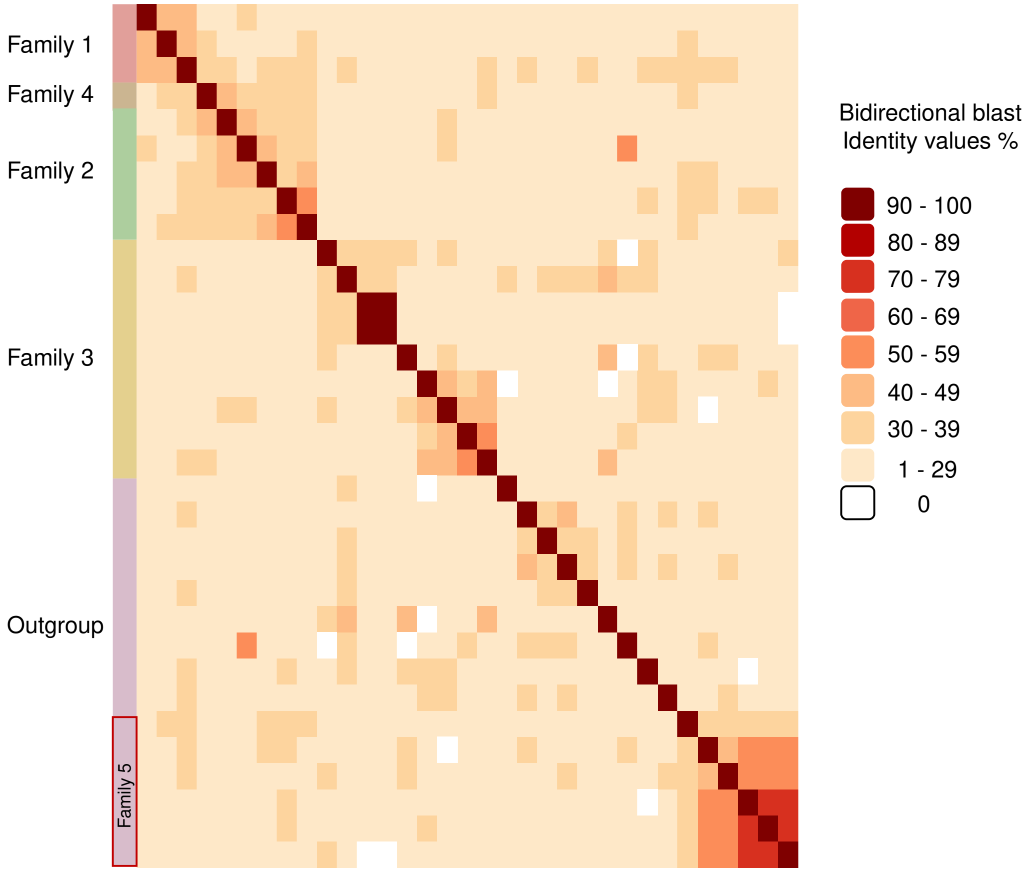

Supplementary Figure S7. Identity values of casposase representative family members. The colored left bar indicates the family according to the colored clades of Figure 6, where family 5 members are indicated inside the red square. The right legend shows the identity value as a percentage.

```
group2 family2 YP 007249538 PPESVQVKTKFAQYRAFEDKKRLQIARQFIDAKFERTHLVLDYLHQRYPDVETDFSSESAKLTKVTTIKELMGVEGVVAV 160
group2 family2 YP 006921765 PPESTNVKTKFAQYRVFEDEKRVKIAKNFIEAKFDKTLVVLDYLKQRYPEIDYNLTEDFEKLKKAKSIKEIMGVEGGVVY 160
group2 family2 WP 023846532 PPESTNVKTRFAQYHAFEDKARIKIAKNFIEAKFDKTQVVLDYLKQRYPEIEYDFSPDLKKLDSAKKINDILGVEGGVAY 160
group2 family2 YP 003542458 PPESTNVKTKFAQYHAFEDQTRVKLAKKFIEAKFDKSIVVLDFLKQRYPDIEFDFSDDVEKLNKTGTIRGLMGVEGGVAW 160
group2 family2 YP 565285    PSESTNVKTKFAQYHAYEDDTRVKLAKKFIEAKFSKSEAVLDYLKLRYPEINYDISVDKGKLENAKSVREILGVEGGVAW 160
group2 family2 YP 565297    PSESTNVKTKFAQYHAYEDDARVKLARKFIEAKFYKSEAVLDYLKQRYPEIEYDFSVDKGKLENAKSVREILGIEGGVAS 160
group2 family2 YP 565305    PSESTNVKTKFAQYHAYEDDVRLKLAKKFIEAKFSKSEAVLDYLKQRDPEIEYDFSDDKAKLEKANSIRDILGVEGGVAW 160
group2 family2 WP 019178002 TPEPKAGGTRLAQYQAFSSERRLEIARKIIDAKIRMSIATLDWMVQRYPELKDELALYQSKLPEADSIAKIRGVEGMVAR 159
group2 family2 YP 003482764 PKEPINGKLKIRQYEIYINKERLKIAEKILEEKIRKSENMLYELSEYYPEIEHIVKKRIEKEEKLPKLSYLLMYEGRVAQ 158
Cas1_1244                   PTSSAHVKTRIKQYEAYYNSKGVEIAKAILNAKIQNQISLLERHG--FDGHKLDLVEKLSKIKGEDIRPKLMGIESRCSK 154
Cas1_1269                   NEDDAHVKTRINQYEAIKNGKGVYTAKQIVLAKILGQNEVLKKYGLRHD--IMGIKEAIGRLEGDTVRKRLMVIEAKASK 154
Cas1_1219                   KEDDSHVATRVAQYKALENGKGLEIAKTIVLSKIEGQNLVLRKYGLELDFSA--LKSRVEALNCDDARYRLNQIEGKASQ 153
Cas1_125                    GDDDAHIRTRIAQYEALKNGKGITIAKQIVLSKIESQNIILKEYSLRHD--LIAIRAKVQSLKSNDIRKRLLPIEGKASE 154
Cas1_938                    RDDDAHVKTRIAQYEALHNGKGIQIAKTIVLSKVESQNMVLKKYGLRLDLAF--FKARIENLNSVDVRRRLLPIEGKASN 153
Cas1_1200                   RDDDSHVKTRIAQYEALKNGKGIEIAKTIVYSKIESQNMVLRKYGLKHDLAV--FKARIEGLNAEHVRRSLLQIEGKASE 153

group2 family2 YP 007249538 HYWDQIQKVIPDREFTSRTNRPHGAADTVNCMLNYGYSLLEAECLRAINSVGLDAHVGYLHEMAIGKNSLAYDFQELFRF 240
group2 family2 YP 006921765 KYWNEFSKAIPEYDFESRSRRATGAGDMVNAMLNYGYALLEAEGLRAINAVGLDPHVGFLHEMTTSKNSLAYDLQEPYRF 240
group2 family2 WP 023846532 KYWNEFSKVIPEYDYESRSRRATGAGDMVNTMLNYGYALLEAECLRTINAVGLDSHVGFLHEMNTGKNSLAYDLQEPFRF 240
group2 family2 YP 003542458 KYWNEFAKAIPAYDFCARYRRPIAAGDKVNVMLNYGYALLEAECLRAINSVGLDPHVGFLHEMNSSKNSLAYDLQEPFRF 240
group2 family2 YP 565285    KYWNEYAKAIPEYDFRARNARASNSGDKVNVMLNYGYALLESECLRAINSVGLDAHVGFLHEMNPSKNSLAYDLQEPFRF 240
group2 family2 YP 565297    KYWNEYSKAIPDYDFRARNARASNSGDKVNVMLNYGYALLESECLRAINSVGLDAHVGFLHEMNPSKNSLAYDLQEPFRF 240
group2 family2 YP 565305    KYWNEYAKAIPEYDFRARNARASNSGDKINVMFNYGYALLESECMRAINSVGLDAHVGFLHEMNPSKNSLAYDLQEPFRF 240
group2 family2 WP 019178002 NYWLIVAETFDKWEFEGRTGRPMAAVDPINALFNYGYSLIEAQCWRAINANGLDPYIGFVHETAPGKSPLAYDLQEPFRW 239
group2 family2 YP 003482764 IYWKELSKIFNKFNFTSRSYSWNNASDEINALLNYSYALLESMIRKHINAVGLDPSIGFLHELASSKTPLVYDLQELFRW 238
Cas1_1244                   VYFGHFKTLFPDFQTPKR--MKYNAEDPLNNLLNLGYEVLKGEVYRAVMYAHLDPYLGYLHSIQFAKPSLVCDIQEVFRG 232
Cas1_1269                   RYFQQIFLLFPKERPDAR--KTFQAYDGINNLFNLGYELLFWKCYRALTKAHLETHLGFMHLTLRGRPSLVCDFEEIYRY 232
Cas1_1219                   HYFSQIFSLFPEKRPERR--VGFKAYDGLNNVFNFGYYVLKCRVYKALLKAKLEPYLGFLHALQNGKPSLACDFMELYRY 231
Cas1_125                    FYFKQVFQLLPKSRIEKR--RGWKAFDGVNNTFNLAYTLLKYRVHSAILKAHLEPYLGFVHGEQFAKPSLVCDFMELYRY 232
Cas1_938                    HYYHEVFRLLPESMVEKR--KGWKAYDGMNNIFNLAYTLLKFRVYSAVLNAHLEPYLGFVHSEQFGKPSLVCDFMELYRY 231
Cas1_1200                   CYFHEVFKLLPNSRIDKR--KTWKAYDGINNTFNLAYTLLKFRVHMAILKVHLEPYLGFIHGEAWSKPSLVCDFMELYRY 231
```

Supplementary Figure S8. Alignment of family 5 casposase members with nine representative casposase genes from family 2 (GenBank accession number indicated in the header). Sequences start in residue 160, intended to show active site residues (in red) proposed by Krupovic et al. [31].

Supplementary Table S1. Metadata of each of the 48 metagenomic data sets used in this study with information about the quality of the assembly.

Supplementary Table S2. Information about the genetic neighborhood (± ORFs) of the 2150 Cas1 used in this study.

Supplementary Table S3. Metadata of the 2015 Cas1 protein sequences used in this study, including the aminoacidic sequence.

Supplementary Table S4. Taxonomic affiliation of the 2980 16S rRNA gene sequences obtained in this study, including the nucleotide sequence.

Supplementary Table S5. PERMANOVA results of Cas1 and 16S rRNA genes according to the marginal and sequential effect of environmental and taxonomic terms.

Supplementary Table S6. Summary of the Cas1 proteins grouped in no reference (NR) clades or casposase clade, showing taxonomy, Cas genes and type of CRISPR-Cas system predicted.
